# The Suitability of *P. falciparum* Merozoite Surface Proteins 1 and 2 as Genetic Markers for *In Vivo* Drug Trials in Yemen

**DOI:** 10.1371/journal.pone.0067853

**Published:** 2013-07-04

**Authors:** Nazeh M. Al-abd, Mohammed A. K. Mahdy, Abdulsalam M. Q. Al-Mekhlafi, Georges Snounou, Nazia B. Abdul-Majid, Hesham M. Al-Mekhlafi, Mun Y. Fong

**Affiliations:** 1 Department of Parasitology, Faculty of Medicine, University of Malaya, Kuala Lumpur, Malaysia; 2 Institute of Biological Sciences, Faculty of Science, University of Malaya, Kuala Lumpur, Malaysia; 3 Department of Parasitology, Faculty of Medicine and Health Sciences, Sana’a University, Sana’a, Yemen; 4 Institut National de la Santé et de la Recherche Médicale, Unité Mixte de Recherche S 945, Paris, France; 5 Université Pierre & Marie Curie, Faculté de Médecine Pitié-Salpêtrière, Paris, France; 6 Resarch Department, University of Science and Technology, Sana’a, Yemen; Johns Hopkins Bloomberg School of Public Health, United States of America

## Abstract

**Background:**

The accuracy of the conclusions from in vivo efficacy anti-malarial drug trials depends on distinguishing between recrudescences and re-infections which is accomplished by genotyping genes coding P. falciparum merozoite surface 1 (MSP1) and MSP2. However, the reliability of the PCR analysis depends on the genetic markers’ allelic diversity and variant frequency. In this study the genetic diversity of the genes coding for MSP1 and MSP2 was obtained for P. falciparum parasites circulating in Yemen.

**Methods:**

Blood samples were collected from 511 patients with fever and screened for malaria parasites using Giemsa-stained blood films. A total 74 samples were infected with P. falciparum, and the genetic diversity was assessed by nested PCR targeting Pfmsp1 (Block2) and Pfmsp2 (block 3).

**Results:**

Overall, 58%, 28% and 54% of the isolates harboured parasites of the Pfmsp1 K1, MAD20 and RO33 allelic families, and 55% and 89% harboured those of the Pfmsp2 FC27 and 3D7 allelic families, respectively. For both genetic makers, the multiplicity of the infection (MOI) was significantly higher in the isolates from the foothills/coastland areas as compared to those from the highland (P<0.05). Pfmsp2 had higher number of distinct allelic variants than Pfmsp1 (20 vs 11). The expected heterozygosity (HE) for Pfmsp1 and Pfmsp2 were 0.82 and 0.94, respectively. Nonetheless, a bias in the frequency distribution of the Pfmsp1 allelic variants was noted from all areas, and of those of Pfmsp2 in the samples collected from the highland areas.

**Conclusions:**

Significant differences in the complexity and allelic diversity of Pfmsp1 and Pfmsp2 genes between areas probably reflect differences in the intensity of malaria transmission. The biased distribution of allelic variants suggests that in Yemen Pfmsp1 should not be used for PCR correction of in vivo clinical trials outcomes, and that caution should be exercised when employing Pfmsp2.

## Introduction

Malaria is a major health problem in Yemen causing 4.9 deaths and 210 disability adjusted life years (DALYs) per 100000 populations [1]. Within the nine countries in the WHO Eastern Mediterranean region, Yemen ranks behind Afghanistan, with 60% of population at high risk of malaria, and is placed among the countries that showed no downward trend in malaria frequency [2], [3]. *Plasmodium falciparum* is the predominant species in Yemen where it is responsible for more than 90% of the malaria cases, the remainder being due to *P. vivax* or *P*. *malariae* [4], [5].

In Yemen, chloroquine (CQ) was used as a first line treatment of uncomplicated malaria. Following the emergence CQ-resistant *P. falciparum* [2], [6], [7], the triple combination of artesunate+sulfadoxine-pyrimethamine has been introduced as a first line antimalarial treatment as a matter of policy. It must be noted that all anti-malarial drugs are available in the Yemeni market and that physicians may not follow the malaria drug policy proposed by the Ministry of Health. Thus, it is crucial to conduct continuous monitoring of anti-malarial drugs efficacy in order to detect the emergence of resistant parasites. Over the last years, anti-malarial drug efficacy trials conducted in endemic areas, rely on polymorphic regions of malarial genes as genetic markers that help classify malaria episodes that occur during the follow-up of patients as recrudescences (treatment failure) or new infections (treatment success) [8]. Two of the most commonly used genetic markers recommended by the WHO for use in such genotyping studies are polymorphic regions within the genes coding for the *P. falciparum* merozoite surface proteins 1 and 2 (MSP1 and MSP2) [9]. The reliability of the genotyping and therefore of the PCR correction of trial outcomes, depends on the discriminatory power of the genotyping, which depends on the extent of allelic diversity and the frequency distribution of the allelic variants of the genetic markers. Thus, if one allelic variant occurs in high frequency in the endemic area, a re-infection with parasites carrying the same allelic variant as the parasites on admission would be misclassified as a recrudescence, i.e., treatment failure. This study was carried out to determine whether the allelic diversity and frequency of the polymorphic regions of the *Pfmsp1* and *Pfmsp2* in *P. falciparum* populations circulating in Yemen is consistent with their use for genotyping studies associated with *in vivo* drug trials.

## Materials and Methods

### Ethics Statement, Study Area and Population

**Figure 1:**
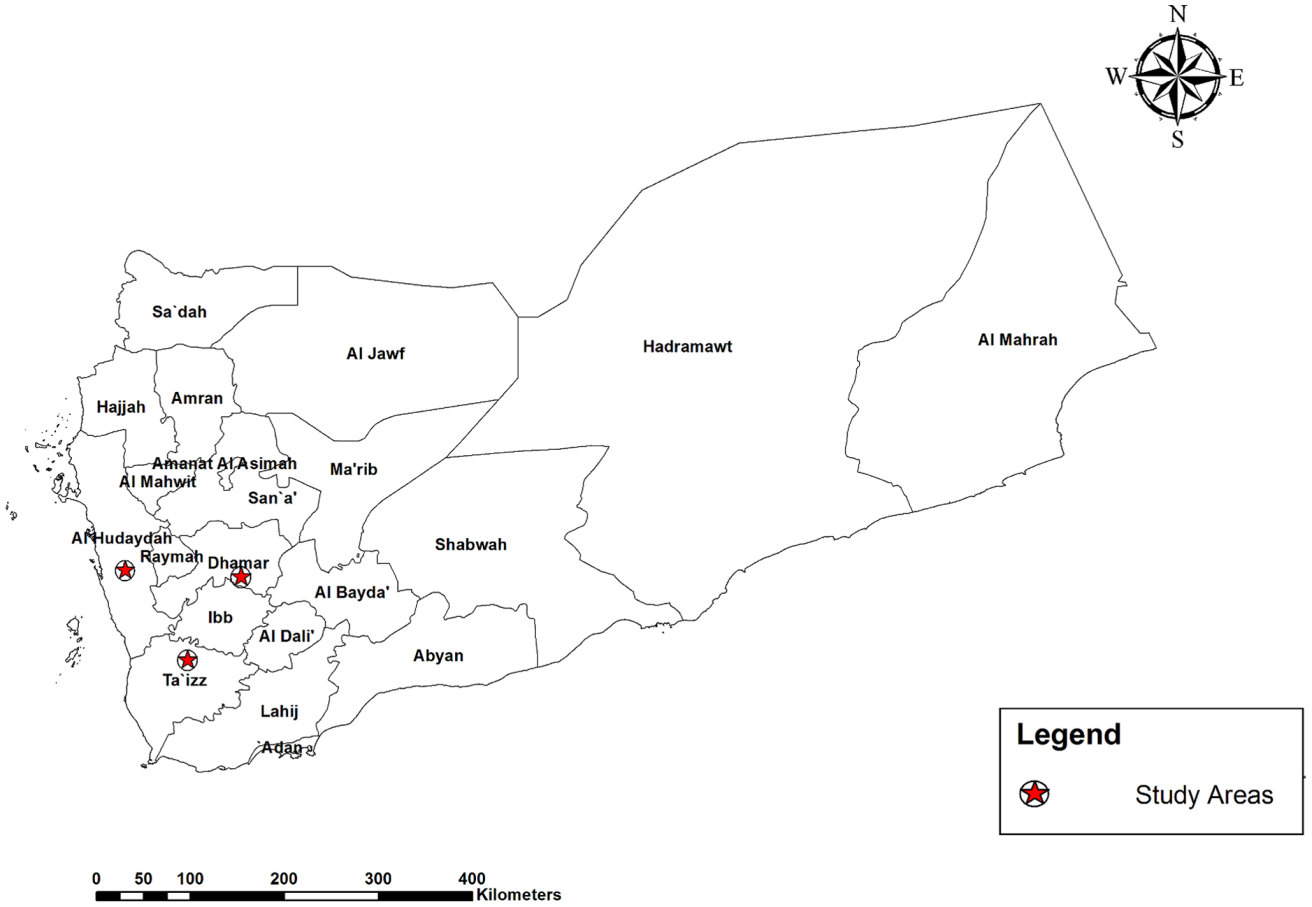
Map of study location.

The present study was conducted in three governorates in Yemen with a total population of 5.9 million [10]. The selected governorates, Taiz, Hodeidah and Dhamar, represented the mountainous hinterland, coastal areas and highland areas, respectively (Figure 1). Living quarters in the rural communities of the study areas are made of mud or stones and have wooden roofs. The majority of the residents are involved in the agriculture, fishery, livestock or handicraft sectors.

The peak time of malaria transmission in the coastal areas occurs in winter (October-April), while in the western mountains, the peak occurs in the summer (May-September). The mountainous hinterland normally shows peak of transmission between October and March. In the highlands areas, which are located at more than 2000 metres above sea level, the transmission occurs throughout the year [4]. *Anopheles arabiensis* is the main vector in the country but *A. culicifacies* plays an important role in the transmission of malaria in the coastal areas. *A. sergenti* has been reported to be a vector in the mountainous hinterland and highland areas [4].

Study subjects were patients with febrile illness attending hospitals and medical centres from June 2008 to March 2009. Patients participated on a voluntary basis after they were given a clear explanation of the research objectives. If the subjects were children, informed consent was obtained from their guardians. As far as we know, the patients had not taken antimalarial treatment prior to admission, but this was not confirmed by further analysis. Patients participated on a voluntary basis after they were given a clear explanation of the research objectives and provided a written informed consent. If the subjects were children, written informed consent was obtained from their guardians. The study protocol was reviewed and approved by the Faculty of Medicine, Sana’a University, Sana’a, Yemen.

### Microscopy

Whole blood from a finger prick was collected from each subject and used to prepare thick and thin blood films. Additional drops of blood were spotted onto Whitman filter paper 3MM (Whatman International Ltd., Maidstone, England), labelled and stored in plastic bags at room temperature until use. Thick and thin blood films were stained with Giemsa stain and examined under microscope at 1000X magnification. The smear was considered negative after screening 100 high power fields. Specimens positive for *P. falciparum* were selected for molecular characterization using MSP1 and 2 markers.

### DNA Extraction and Molecular Analysis

Parasite genomic DNA was extracted from a 50 µl blood spot on the filter paper. Briefly, a disc was punched out from the blood spot using a pre-flamed paper puncher and placed in a 1.5 ml centrifuge tubes using a pair of sterile forceps. DNA was extracted using QIAgen DNA Mini Kit blood and tissue (QIAGEN, Cat. no. 51306, Germany) according to the manufacturer’s instructions, and 100 µl of template were obtained.

Allelic families of *Pfmsp1* (block 2) and *Pfmsp2* (block 3) were analysed using nested PCR assays as published previously [11]. Primary PCR was carried out using primers specific for the two loci. Secondary PCR was carried out individually for each of the primers pairs specific for allelic families of *Pfmsp1* (K1, MAD20 and RO33) and for those of *Pfmsp2* (FC27 and 3D7/IC). Primary PCR was performed in 50 µL reaction mixture containing 5 µL of DNA template, 1X i-Taq™ buffer free of MgCl_2_ (iNtRON BIOTECHNOLOGY, Seoul, Korea), 1 mM of MgCl_2_ (iNtRON BIOTECHNOLOGY, Seoul, Korea), 125 µM dNTP (iNtRON BIOTECHNOLOGY, Seoul, Korea), 0.25 µM of each primer and 1.25 U of i-Taq™ DNA polymerase (iNtRON BIOTECHNOLOGY, Seoul, Korea). Secondary PCR was performed in 25 µL reaction mixture containing 3 µL of DNA template and the same concentrations as the primary PCR. In both amplifications, samples were incubated in the MyCycler thermal cycler (Bio-Rad, Hercules, USA) under the following conditions: initial denaturing step at 95°C for 5 min, annealing at 58°C for 2 min and extension at 72°C for 2 min, followed by 24 cycles of denaturing for 1 min at 94°C, annealing for 2 min at 58°C and extension for 2 min at 72°C, followed by a final annealing at 58°C for 2 min and a final extension at 72°C for 5 min. The PCR products were subjected to electrophoresis in 2% agarose gels and stained with Syber green. The sizes of the *Pfmsp1*and *Pfmsp2* allelic variants were measured using Quantity One software (Bio-Rad, USA). Two allelic variants whose sizes differed by 25 bp or less were placed in the same bin size and considered to belong to the same allelic variant.

### Definitions

The detection of a single PCR fragment of an allelic family was considered as an infection with one genotype. Multiple infections of *P. falciparum* were defined as the presence of more than one genotype of either *Pfmsp1*or *Pfmsp2* in a single blood sample [12]. The multiplicity of infection (MOI) is the mean of the frequency of the multiple infections in a single blood sample i.e., the total of genotypes divided by the total number of samples [12]. MOI was calculated for *Pfmsp1* and *Pfmsp2*. Rural area was defined as an area outside cities and towns. Cities and towns were considered as urban areas. Parasitaemia was graded as low (1–999/µl), Moderate (1000–9999/µl) and high (>10000/µl).

### Data Analysis

Data were analysed using the SSPS programme for Windows version 11.5 (SPSS Inc., Chicago, IL, USA). The associations between proportions were tested using χ^2^ test. Non-parametric statistical tests (Mann-Whitney Test and Kruskal Wallis Test) were used to test the difference in MOI between areas, age groups and parasite densities. *P*<0.05 was considered statistically significant. Heterozygosity was calculated by the formula H_E_ = [n/(n –1)][(1-∑Pi^2^)].

## Results

### Multiplicity of Infection and Distribution of Allelic Families

The study was carried out on 511 febrile patients seeking health care. Of these patients, 74 had *P. falciparum* infection and were selected for the genotyping study. The patients’ characteristics are indicated in table 1. Positive amplification of *Pfmsp1* was only observed in 57 (77%) and of *Pfmsp2* in 56 (76%) of the samples, for both markers in 42 samples, and failure to amplify either marker was noted in only 3 samples, (Table 2). In these all three *Pfmsp1* allelic families (K1, MAD20 and RO33) and the two *Pfmsp2* allelic families (FC27 and 3D7) were detected (Table 2). For *Pfmsp2*, the 3D7 allelic family was found more frequently than that of FC27 (89% vs 55%) in the samples, while the *Pfmsp1* allelic families, (K1 MAD20, and RO33) were observed in 58%, 28% and 54% of the samples, respectively. The frequencies of mixed infection in which two allelic families from *Pfmsp1* or both the allelic families of *Pfmsp2* were found were 43%. The *Pfmsp1* K1 allelic family was significantly more frequent in isolates from the highlands, whereas the *Pfmsp1* MAD20 and RO33 and the *Pfmsp2* FC27 allelic families were significantly more frequent in those from the foothills/coastland areas. Of 57 isolates positive for *Pfmsp1*, 22% harboured mixed genotypes, principally with one variant from two of the three allelic families. For *Pfmsp2*, 45% of the isolates harboured mixed genotypes, again principally with one variant from each of the two allelic families. The isolates that harboured mixed genotypes were principally those collected from foothills/coastland areas (Table 2). In only six of the samples with mixed genotypes (all collected from the foothills/coastland areas) were multiple variants from the same allelic family observed (four samples had 2 *Pfmsp2* FC27 variants of which one had also two *Pfmsp1* MAD20 variants, and 2 samples had 2 *Pfmsp2* 3D7 allelic variants). The overall multiplicity of infection (MOI) was 1.4 for *Pfmsp1* and 1.6 for *Pfmsp2*. The MOI for the two markers did not vary with age, though there was a tendency for higher MOIs with increasing parasitaemia (Table 3). Patients with high parasitaemias were equally distributed between the highland and foothills/coastland samples, 24 and 23, respectively.

**Table 1:** Characteristics of patients.

Variables*	No (%)
**Study area**	
Foothills/Coastland	38 (51)
Highlands	36 (49)
**Gender**	
Male	43 (63)
Female	25 (37)
**Age**	
<5 years	19 (28)
5–10 years	9 (13)
>10 years	40 (59)
**Parasitaemia**	
Low	4 (6)
Moderate	16 (24)
High	47 (70)

* The characteristics of 6 patients were missing except the study location. The parasitaemia was missing in 7 patients.

**Table 2:** DNA amplification success rate and distribution of allelic families of MSP1 and MSP2 and infection with mixed families in Yemen *P. falciparum* isolates.

		Highland	Foothills/coastland	Total	?^2^	P value
**No. examined**	n	36	38	74		
**Failures (** ***Pfmsp1*** ** & ** ***Pfmsp2*** **)**n (%)		3	0	3		
**Success (** ***Pfmsp1*** ** & ** ***Pfmsp2*** **)** **n (%)**		19 (58)	23 (61)	42 (59)		
***Pfmsp1*** Positives n (%)		26 (72)	31 (82)	57 (77)		
***Pfmsp1*** ** n (%)**	K1	22 (85)	11 (35)	33 (58)	14	<0.05
	MAD20	2 (8)	14 (45)	16 (28)	9.8	<0.05
	RO33	6 (23)	25 (81)	31 (54)	18.9	<0.05
Mixed *Pfmsp1*		4 (15)	18 (58)	22 (39)	10.9	<0.05
***Pfmsp2***Positives n (%)		26 (79)	30 (79)	56 (76)		
***Pfmsp2*** ** n (%)**	FC27	9 (35)	22 (73)	31 (55)	8.5	<0.05
	3D7/IC	21 (81)	29 (97)	50 (89)	3.8	0.055
Mixed *Pfmsp2*		4 (15)	21 (70)	25 (45)	17	<0.05
						
Mixed Genotypes		8 (24)	29 (76)	37 (52)		

**Table 3:** Distribution of MOI of P. falciparum according to study area, age and parasitaemia.

	MSP1		MSP2	
Variables	MOI	Significance	MOI	Significance
**Study area** *				
Foothills/Coastland	1.7	P<0.01	1.9	P<0.01
Highlands	1.2		1.2	
**Age** #				
<5 years	1.4	P>0.05	1.7	P>0.05
5–10 years	1.6		1.6	
>10 years	1.5		1.5	
**Parasitaemia** #				
Low	1.0	P>0.05	1.3	P>0.05
Moderate	1.3		1.4	
High	1.6		1.7	

* Mann-Whitney Test was used.

# Kruskal Wallis Test was used.

### Allelic Frequency and Distribution

**Figure 2:**
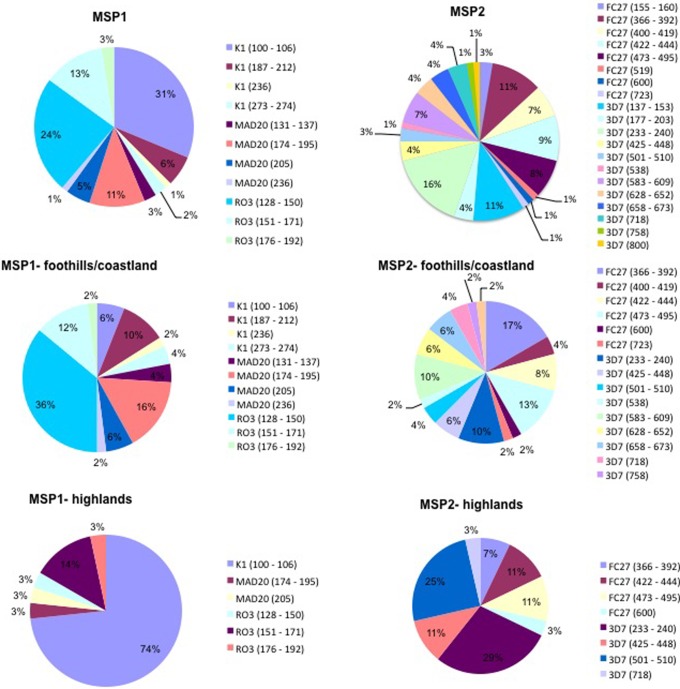
Allelic diversity and frequency of alleles of MSP1 and MSP2 in general and in two endemic areas, foothills/coastland and highlands in Yemen.

The distribution of the *Pfmsp1* (block 2) and the *Pfmsp2* (block 3) allelic variants in the different geographical areas is summarised in Figure 2. *Pfmsp2* had higher number of distinct alleles (n = 20) than *Pfmsp1* (n = 11). Within the *Pfmsp2* allelic families, a total of 12 and 8 allelic variants were observed for the 3D7 and FC27 families, respectively. The *Pfmsp1* allelic families K1 and MAD20 had each 4 distinct allelic variants and RO33 had three. The highest diversity of allelic variants for both loci was noted in samples from the foothills/coastland areas. The expected heterozygosity (H_E_) was high for *Pfmsp2* in the parasites collected from all areas (0.83–0.93), but for *Pfmsp1* the H_E_ was high in the foothills/coastland samples (0.82) but it was significantly lower (0.44) for the samples collected from the highland areas (Table 4).

**Table 4:** Genetic diversity of *msp1* and *msp2* expected in heterozygosity (HE) H_E_ = [n/(n-1)] [(1-ΣPi^2^)].

	MSP1	MSP2
**Foothills/coastland**	0.82	0.93
**Highland**	0.44	0.83
**All areas**	0.82	0.94

## Discussion

To our knowledge, this is the first study that examined the genetic diversity of *P. falciparum* in Yemen using the polymorphic regions of the genes coding for MSP1 and MSP2. We confined the analysis to symptomatic infections, since the aim was to establish the usefulness of these genotyping markers in the context of *in vivo* drug trials. Representatives of all the allelic families (K1, MAD20, RO33 for *Pfmsp1*; FC27 and 3D7 for *Pfmsp2*) were found. None of the *Pfmsp1* and *Pfmsp2* allelic families was exclusively restricted to a particular geographical location in Yemen, though the frequency of some was significantly higher in one area as compared to another. Similar types of *Pfmsp1* and *Pfmsp2* allelic families distribution have been reported from areas of diverse endemicities such as French Guiana [13], Kenya [14], Peru Myanmar [15] and Pakistan [16]. Meiotic recombination during the sexual reproduction stage is a major mechanism for the generation of genetic diversity of *P. falciparum*. The prevalence of mixed infections ranges from 20–30% in areas of low endemicity [17], [18], 50% in mesoendemic regions [19], [20] to 100% in some holoendemic areas [17], [21]. In the samples collected in this study, mixed genotype infection with more than one genotype were observed in 58% of the samples Thus, the high prevalence of mixed genotype infection could enhance cross-fertilization resulting in the generation of novel alleles [22].

We are aware that the total number of samples analysed is relatively low, and that sample numbers for the different areas are possibly too low for robust statistical analyses. A positive association between MOI and level of transmission of *P. falciparum* has been well documented [17], [23]–[25], though some exceptions where a high MOI is observed in low endemic areas are known [26], [27]. The MOI of *P. falciparum* infections in our samples was 1.4 for *Pfmsp1* and 1.5 for *Pfmsp2*. The highest MOI was observed for parasites collected in the foothills/coastland areas and the lowest for those collected in the highland areas. These data suggest that level of malaria transmission is different in the two locations. The MOI recorded for the foothills/coastland samples was similar to those observed in hyperendemic areas in Africa [17], [24], [28], while that recorded for the highland samples was closer to those reported from low transmission areas such as in central and Southeast Asian countries [16], [23], [29].

A high number of allelic variants were observed for *Pfmsp1* (11) and *Pfmsp2* (20) in the Yemeni *P. falciparum* populations. The allelic diversity was higher in the foothills/coastland samples as compared to those from the highlands, and higher in the rural samples as compared to the urban samples. This is consistent with the MOI data and is likely to be related to differences in the intensity of the malaria transmission. Whereas the overall high allelic diversity of *Pfmsp1* and *Pfmsp2* indicates that these genetic markers would have sufficient discriminatory power to help classify recurrent episodes during follow-up of treated patients into recrudescences or re-infections, a bias in the frequency distribution was noted for the allelic variants of both *Pfmsp1* and *Pfmsp2* when each specific area was considered separately. This was particularly clear for the parasites in the highlands where 74% of the *P. falciparum* isolates carried a single *Pfmsp1* K1 allelic variant. Thus, if one uses the *Pfmsp1* marker to distinguish recrudescences from re-infection in this area, about seven times out of ten a true re-infection will be misclassified as a recrudescence. This is an unacceptably high level of misclassification of re-infections as recrudescences, which would overestimate the prevalence of anti-malarial resistant parasites. In the highland parasite populations, where *Pfmsp2* 3D7 allelic variants were also present at high frequency (29% and 25%), which again is likely to confound classification into recrudescences or re-infections. These findings support the long-standing recommendations from WHO to genotype multiple loci in order to distinguish recurrent parasitemias. To our knowledge, PCR genotyping was associated to a single *in vivo* clinical trial in Yemen, conducted at the end of 2002 to assess the efficacy of chloroquine [30]. In that study, treatment failure was recorded in 74 of the 122 patients (60%) that were enrolled and followed-up during 14 days (trial protocol according to WHO monographs WHO/CDS/CSR/EPH/2002.7 and HO/CDS/RBM/2002.29.). However, genotyping based on *Pfmsp2* alone was conducted on paired samples from only 24 of the treatment failures, leading to classify 17 as recrudescences. Although there is the possibility that this CQ trail was biased by their reliance upon a single marker, this is speculative, and the lack of integrity of their data is manifest anyway.

In conclusion, the study indicated that although the *P. falciparum* populations in Yemen are genetically diverse with respect to *Pfmsp1* and *Pfmsp2*, the extent of this diversity varies geographically. In highland areas, the markedly biased allelic frequency distribution precludes the use of *Pfmsp1* and is likely to reduce the utility of the *Pfmsp2* as suitable markers for genotyping in the context of *in vivo* drug trials. Thus, in Yemen, the third *Pfglurp* (glutamate-rich protein) recommended for these genotyping studies must be included in the analysis provided its suitability (i.e. a lack of biased allelic variant frequency distribution) is demonstrated. Alternatively, microsatellite-based genotyping [31] or SNP bar-coding [32] should be considered and assessed for use in conjunction with *in vivo* drug trial to be conducted in Yemen.
